# Hunger enhances consistent economic choices in non-human primates

**DOI:** 10.1038/s41598-017-02417-5

**Published:** 2017-05-24

**Authors:** Hiroshi Yamada

**Affiliations:** 1 National Center of Neurology and Psychiatry, 4-1-1 Ogawa-Higashi, Kodaira, Tokyo, Japan; 2 Division of Biomedical Science, Faculty of Medicine, University of Tsukuba, Tsukuba, Ibaraki, Japan; 3 Graduate School of Comprehensive Human Sciences, University of Tsukuba, Tsukuba, Ibaraki, Japan

## Abstract

Hunger and thirst are fundamental biological processes that drive consumption behavior in humans and non-human animals. While the existing literature in neuroscience suggests that these satiety states change how consumable rewards are represented in the brain, it remains unclear as to how they change animal choice behavior and the underlying economic preferences. Here, I used combined techniques from experimental economics, psychology, and neuroscience to measure food preferences of marmoset monkeys (*Callithrix jacchus*), a recently developed primate model for neuroscience. Hunger states of animals were manipulated by scheduling feeding intervals, resulting in three different conditions: sated, non-sated, and hungry. During these hunger states, animals performed pairwise choices of food items, which included all possible pairwise combinations of five different food items except for same-food pairs. Results showed that hunger enhanced economic rationality, evident as a decrease of transitivity violations (item A was preferred to item B, and B to C, but C was preferred to A). Further analysis demonstrated that hungry monkeys chose more-preferred items over less-preferred items in a more deterministic manner, while the individual food preferences appeared to remain stable across hunger states. These results suggest that hunger enhances consistent choice behavior and shifts animals towards efficient outcome maximization.

## Introduction

There is a growing consensus in neuroscience that brain networks involved in economic decision making are strongly influenced by hunger and thirst^1–3^. These neural mechanisms suggest that hunger and thirst affect economic decision makings. Existing behavioral studies have examined the effects of hunger states on risk preferences in a wide range of species^4–9^, primarily focusing on how hunger governs behavior that reduces the risk of starvation. In contrast, less headway has been made toward understanding how hunger states affect the underlying preferences of food items.

In the economic literature, theoretical and empirical frameworks for examining preferences and rational choice behavior have been provided specific to humans^10–14^. At the theoretical level, a logically consistent chooser behaves as if he consults an internal preference ranking of items (i.e., utility), and then, a choice is processed to maximize benefits according to these internal preferences. These economic frameworks have been used in noneconomic work in animals, with the aims of understanding to what extent animals and humans share degrees of economic rationality^5, 15^. For example, transitivity of preferences has been widely used as a fundamental marker of the rationality of economic preferences^10^. That is, if A is preferred to B, and B to C, then A is preferred to C. However, empirical studies have indicated that both humans and animals violate this principle somewhat^10, 16–21^. While humans and animals thus do not conform to some models of economic rationality, it remains unclear how hunger states relate to transitivity violations and economic rationality.

Another line of inquiry in neuroscience and psychology has examined the effects of satiety selective to a particular food item (i.e. taste) on consumption behavior, known as sensory-specific satiety or reinforcer devaluation^22, 23^. For example, after satiation with a particular food item, consumption of the satiated food item is suppressed, which suggests that the value of the specific food item decreases while the values of other foods do not^24^. The neural activity involved in this devaluation has been suggested to be distributed across the central nervous system^25–27^, but these studies do not highlight the overall effect of hunger on food preferences in the economic sense. Similarly, a small number of behavioral studies in human nutrition science have found that when subjects are severely deprived of food, the choice of food items changes^28^. While this suggests that food preferences change depending on hunger state, other factors, such as the tradeoff between portion size and delivery delay, may strongly affect food choices^29, 30^. As of yet, existing work has not fully investigated whether food preferences are consistently maintained across different hunger states. Therefore, it is important to determine the effect of hunger states on food preferences by using standard techniques from human experimental economics, i.e. transitivity of preferences.

In the present study, I investigated the following two questions: (i) Are preferences for food items in non-human primates stable across distinct hunger states? (ii) Are patterns of choice behavior maintained consistently across hunger states from an economic point of view? To this aim, I used standard economic techniques combined with psychological and neuroscientific ones to quantify item preferences of marmoset monkeys in different hunger states. The results suggest that hunger enhances consistent choice behavior and shifts animals towards efficient outcome maximization.

## Results

**Figure 1:**
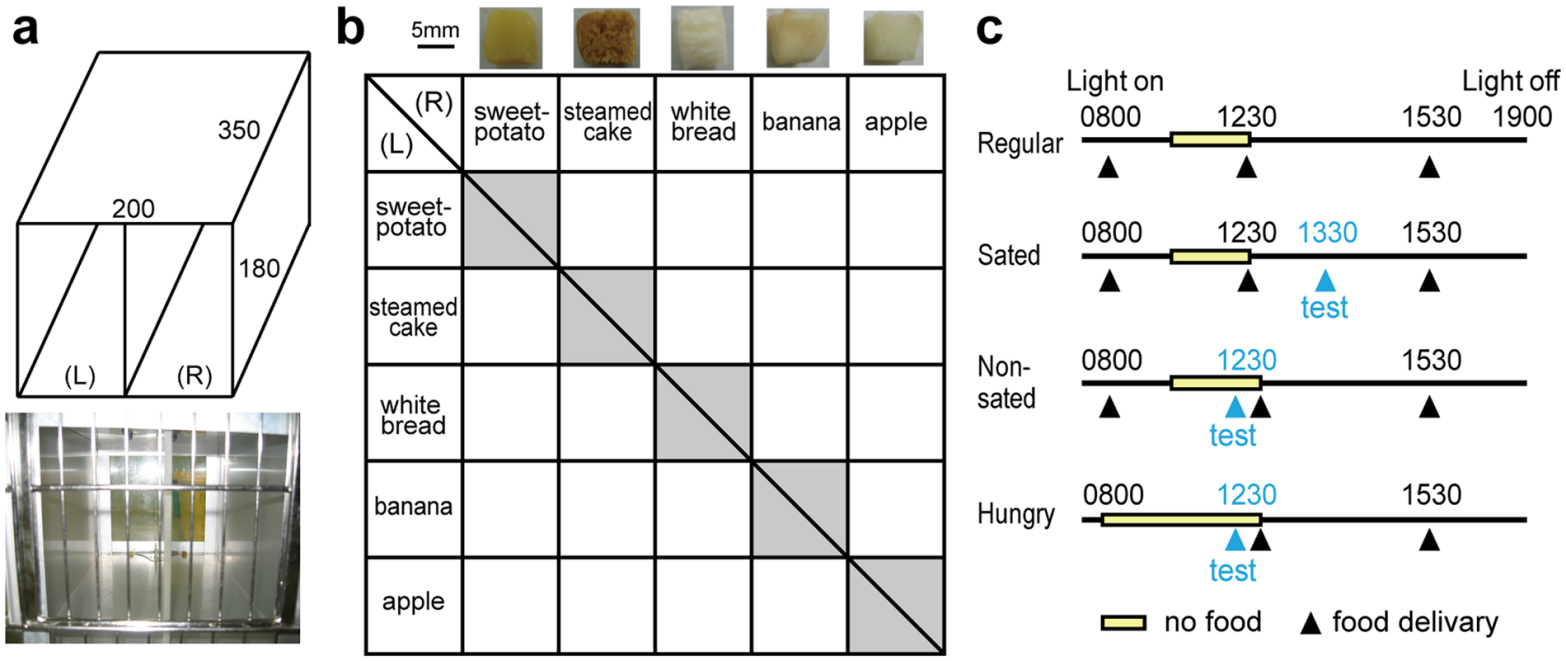
Experimental setting. (**a**) A schematic drawing (top) and picture (bottom) of the experimental chamber. During the pairwise food choice test, a two-armed chamber was attached to the home cage of animals. Values in the figure indicate mm. Two small food items were located at the far end of the two compartments (left/right). (**b**) Match-up matrix of the five food items with left-right alternation. All pairs (except same food pairs) were used for the pairwise choice test. Pictures of the food items are represented above the matrix with 5 mm scale bar. (**c**) Daily meal schedules in the regular condition and the three controlled food access conditions (sated, non-sated, and hungry) during the pairwise food choice test.

Six marmoset monkeys performed a pairwise food choice test in an experimental chamber attached to their home cage (Fig. 1a). In this task, pairwise combinations of five food items (not including same-food pairs) with left-right alternation were presented, and each monkey chose between the two presented food items (Fig. 1b, 20 choices). To minimize the potential change of hunger state during testing, monkeys made only two choices of a given food pair per day. These 20 choices were made under three controlled food access conditions: sated, non-sated, and hungry (Fig. 1c), resulting in a total of 60 choices over 30 testing days in each monkey.

### Preferences for food items

**Figure 2:**
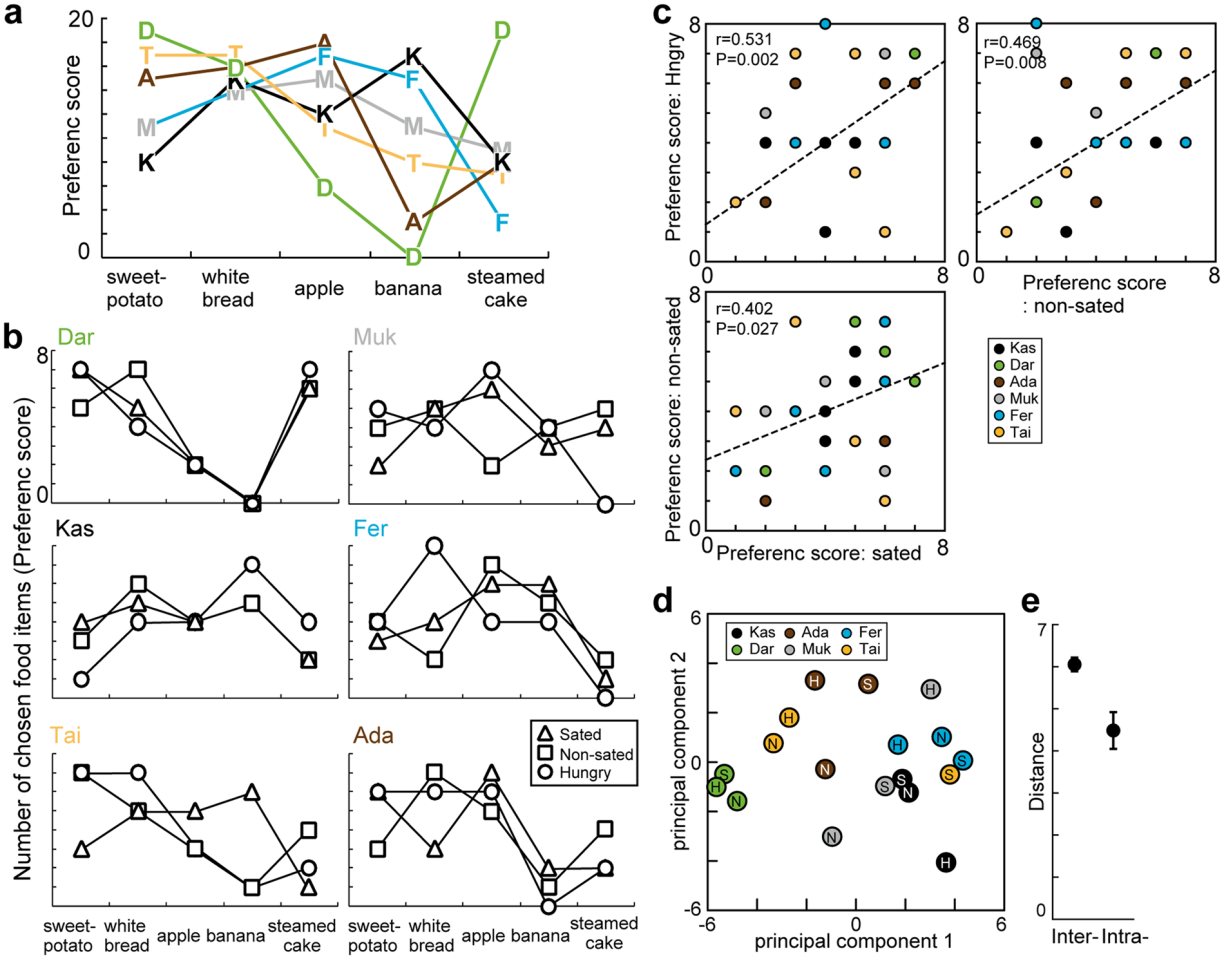
Individual food preferences inferred from the animals’ choices. (**a**) The preference score, defined as the number of chosen food items, was plotted for the six monkeys. Aggregated data of three food access conditions were plotted for each monkey. The possible score ranged from zero to 24 for 60 choices. Characters in the figure indicate individual monkeys (A:Ada, D:Dar, F:Fer, K:Kas, M:Muk, and T:Tai). (**b**) Same as **a**, but for the preference score in each of three food access conditions in the six monkeys. The possible score ranged from zero to eight for 20 choices in each of the three food access conditions. (**c**) Pairwise plots of the preference scores in the three food access conditions. Dashed lines indicate regression slopes. Correlation coefficients and statistical significance are shown. (**d**) Comparison of the preference scores between and within monkeys. The preference scores in each condition in each animal were plotted in Euclidean space using principal component analysis. S: sated condition, N: non-sated condition, H: hungry condition. (**e**) Plot of the averaged interindividual and intraindividual distances. Error bars indicate the S.E.

I first visualized overall tendency of the food item preferences in each individual monkey irrespective of the food access conditions. The choices made by each animal in the pairwise food choice test were used to infer the preferences for food items, as in a previous neuroscientific study^31^. The frequency of chosen food items was used to calculate preference scores for each item. Aggregated data across three food access conditions (sated/non-sated/hungry) indicated that individual monkeys seemed to have a specific pattern of preference scores (Fig. 2a). For example, monkey Dar frequently chose sweet potato, white bread, and steamed cake, but never chose banana. In another example, monkey Kas frequently chose white bread and banana, but less frequently chose sweet potato and steamed cake.

I quantitatively examined whether the individual preferences changed depending on the hunger states by comparing the pattern of preference scores between three food access conditions (Fig. 2b). Irrespective of the condition, individual monkeys showed similar patterns of preference scores between three food access conditions, that were clearly observed in monkeys Dar and Kas, but was not very clear in monkey Tai. The preference scores for food items in each individual were not significantly different between the three food access conditions (Chi-squared test, P > 0.18 for all monkeys). Additionally, variances of the preference scores were not significantly different between three food access conditions (F-test, P > 0.16 for all conditions). Thus, the preference scores for food items did not differ according to the different hunger states.

To further examine the similarity of the preference scores between the three food access conditions, correlation coefficients for the preference score were estimated for each pair of the three food access conditions (Fig. 2c). Aggregated data across monkeys indicated that preference scores in the three food access conditions were moderately correlated with each other; correlation coefficients were within the range of 0.40–0.53 (sated vs. non-sated: r = 0.40, P = 0.03; non-sated vs. hungry: r = 0.47, P = 0.008; hungry vs. sated: r = 0.53, P = 0.002). Thus, the overall preference scores for food items were similar across hunger states.

To further examine individual preferences for food items, the preference score was analyzed using principal component analysis^32^, in which the similarity of the preference scores was evaluated as interindividual and intraindividual Euclidean distances in principal component space for all conditions (Fig. 2d and e). The analysis showed that the pattern of preference scores of a given animal were positioned closely in the Euclidean space (Fig. 2d), and suggested that the pattern of the preference scores were similar between the three food access conditions in each monkey. Indeed, intraindividual Euclidean distances were significantly smaller than those of interindividual distances (Fig. 2e, two sample t-test, P = 0.002). Thus, intraindividual variation of the preference scores between hunger states was smaller compared to interindividual differences.

In short, these analyses of preference scores indicated that individual monkeys’ food item preferences appeared not differ between hunger states.

### Effect of hunger states on monkeys’ food consumption

I examined the effect of the food access condition on monkeys’ consumption behavior by analyzing the number trials in which monkeys did not eat either food item. Analysis of the aggregated data across monkeys showed that number of the ‘uneaten’ trials was significantly greater in the sated and non-sated conditions compared to the hungry condition (sated: 12/120 (10%); non-sated: 12/120 (10%); hungry: 1/120 (0.8%), Chi-squared test, P < 0.006). In the individual-based data analysis, there was no statistically significant differences, but the same tendency seemed to appear on average (mean and S.E. of six monkeys, sated: 2.0 ± 1.63; non-sated: 2.0 ± 1.03; hungry: 0.17 ± 0.17, one-way ANOVA, P = 0.43). This was because individual monkeys showed distinctive responses. Namely, only three monkeys sometimes did not eat either food in the sated or non-sated conditions. In these uneaten trials, monkeys sometimes showed very slow responses or did not enter the testing chamber. In contrast, such behavioral responses were observed only once in the hungry condition. In the other three monkeys, however, the uneaten trials were never observed in any food access condition. Overall, this result could indicate either of the following two possibilities: i) the effect of hunger states on monkeys’ consumption behavior was dependent on each individual, and/or ii) the effect was subtle and was not detected on monkeys’ consumption behavior during testing because the sample size was not large enough.

### Structure of the preferences and transitivity violations

**Figure 3:**
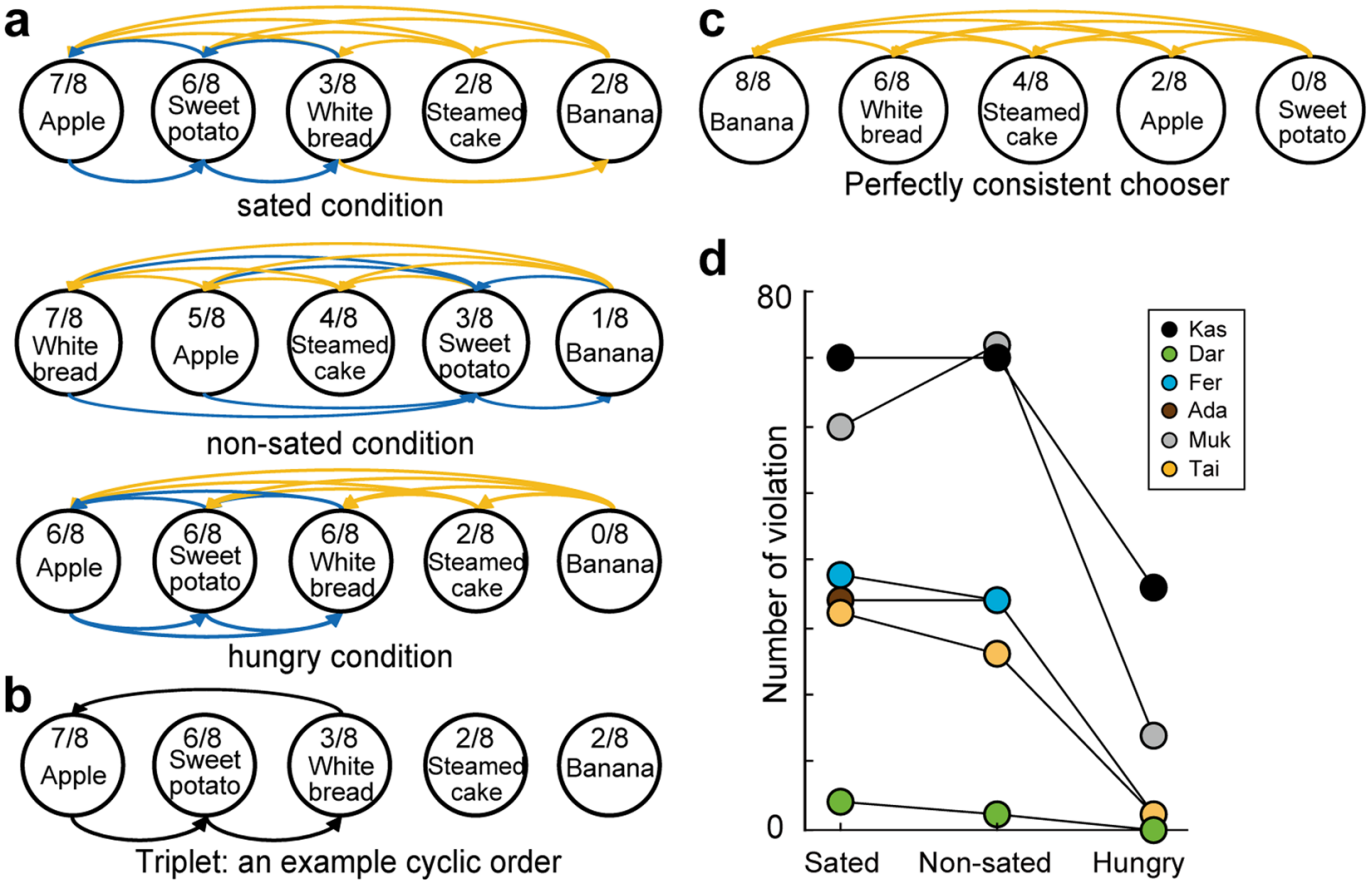
Transitivity violations decreased when monkeys were hungry. (**a**) The structure of preferences in monkey Ada in the three food access conditions. The items are ordered left-to-right according to the preference scores. Arrows are connected from an unchosen item to a chosen item. Yellow arrows indicate that the monkey chose one item over another item twice. Blue arrows indicate split choices (chose each item once). (**b**) An example of the transitivity violation in a triplet in monkey Ada in the sated condition. (**c**) Example choices made by a hypothetical chooser with perfectly consistent behavior. (**d**) The number of transitivity violations equal to or larger than triplets.

Next, I determined the structure of the preferences inferred from the pairwise comparisons according to traditional economic analyses, in which transitivity of preferences (if item A is preferred to item B and B is preferred to C, then A is preferred to C) and its violations were examined. For each food access condition and for each monkey, I first visualized all choices among all pairs. An example of this visualization is provided for monkey Ada in the three food access conditions (Fig. 3a). In the sated condition, for example, she chose one item over another item twice for most of comparisons (yellow arrows). In two cases (apple vs. sweet potato and sweet potato vs. white bread), however, she chose each item once (blue arrows, split choices). In the non-sated and hungry conditions, a small number of the split choices were also occasionally observed. In this example in the sated condition, a cyclic order of items was observed in several cases, which is a hallmark of a transitivity violation: i.e., item A was preferred to item B and B was preferred to C, but C was preferred to A (an example is shown in Fig. 3b). No such cyclic order of items should exist if a subject has strict item preferences and makes choices to maximize his benefit perfectly, in which if A was preferred to B and B was preferred to C, then A should be preferred to C all the time (Fig. 3c).

I quantitatively examined how frequently transitivity violation appeared in distinct hunger states. All possible triples were 80 (_5_C_3_ × 2^3^) in this analysis: i.e., all possible combination of three items out of five items (_5_C_3_) and each pairwise comparison of three items were made twice (2^3^). In the example animal Ada, the number of cyclic orders among three items (intransitive triples) was 14 out of 80 (17.5%) in the sated condition. On average, intransitive triples were rarely observed in the hungry condition (mean and S.E., sated: 15.8 ± 2.7%; non-sated: 15.4 ± 3.6%; hungry: 5.4 ± 2.4%, one-way ANOVA, P = 0.039), which suggests that hungry monkeys largely maintained transitivity of food preferences. The analysis was extended for all possible cycles larger than triples. The total number of combinations equal to or larger than triples was 192 $({}_{5}{\rm{C}}_{3}\times {2}^{3}+{}_{5}{\rm{C}}_{4}\times {2}^{4}+{}_{5}{\rm{C}}_{5}\times {2}^{5})$ in this analysis. Again, transitivity violations were rarely observed in the hungry condition (Fig. 3d, one-way ANOVA, P = 0.047). These results suggest that monkeys were mostly consistent in their choices when they were hungry, and less consistent when they were not hungry.

**Figure 4:**
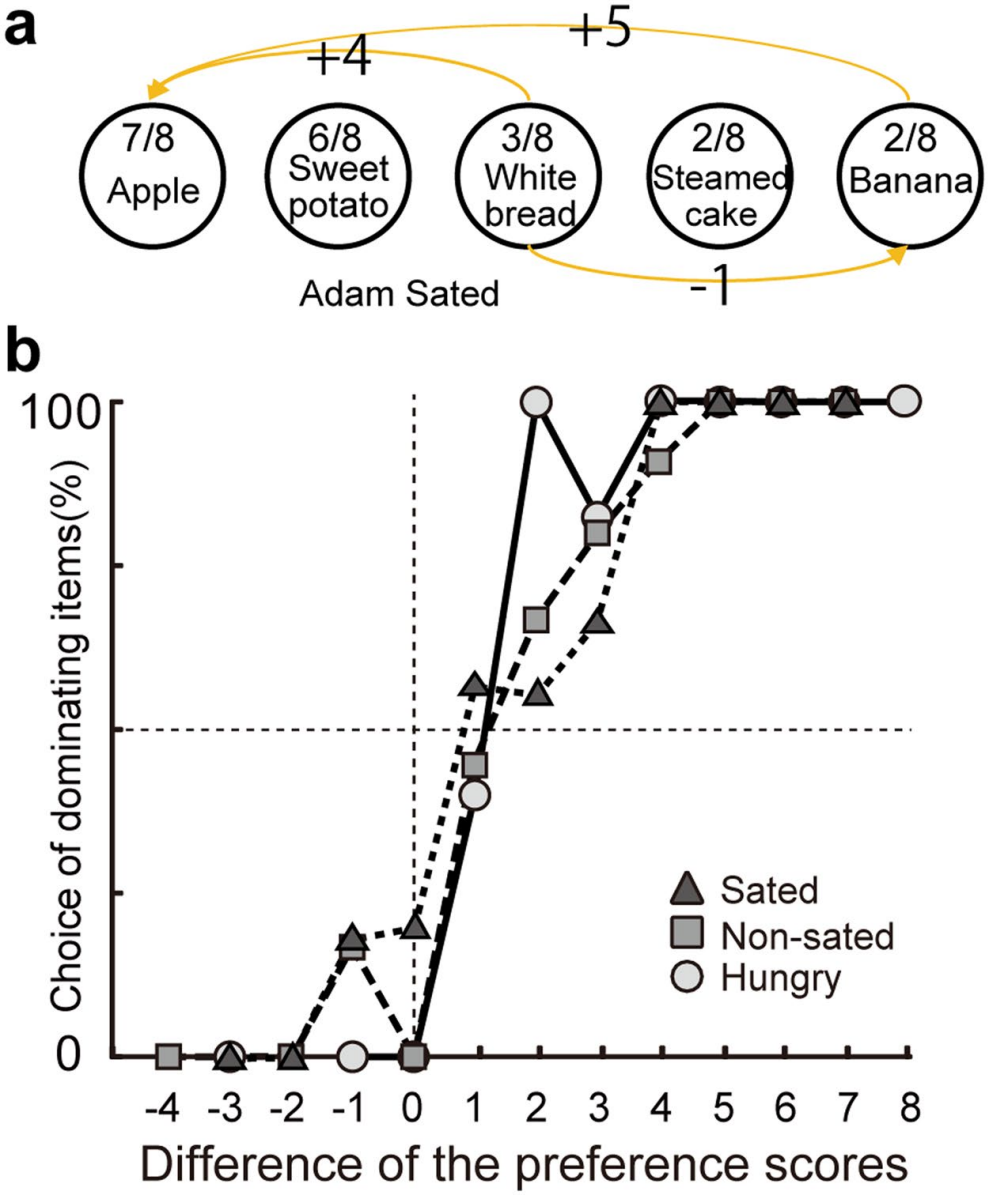
Effect of hunger states on the choice of dominating food items. (**a**) Three example choices of the dominating items as a function of the difference of preference scores in monkey Ada in the sated condition. Values on the yellow arrows indicate differences in the preference score between two items. (**b**) Percent choices of the dominating items as a function of the difference of preference scores in each of the three food access conditions. Data of all animals were aggregated in each of the three food access conditions.

To further examine the decision mechanism behind these hunger state-dependent transitivity violations, I examined how the differences of the preference scores (i.e. value differences among items for comparisons) are related to the food choices, using a method analogous to psychometric analysis^33^. In this analysis, I detected the choice of dominating items (items chosen over another twice, i.e., 100% of the time in a given pair) and used this as an indicator of consistent behavior, because increases in the choice of dominating items result in decreases in transitivity violations. I then examined their relationship with the difference of the preference scores (three examples are shown for monkey Ada in the sated condition in Fig. 4a). The analysis showed that in each of three food access conditions, the choice of dominating items increased as the difference of preference scores increased (Fig. 4b, generalized linear model, P < 0.001). Importantly, monkeys’ hunger states had a significant effect on the choice of dominating items. The choice curves for dominating items were steeper in the hungry condition and became shallower in the non-sated and sated conditions (generalized linear model, P = 0.032). Thus, hunger states appeared to profoundly affect the economic choices derived from the preference differences. This result suggests that hunger decreased the level of stochasticity and shifted animals to choose preferred items more consistently.

### Effect of hunger states on equivalence relations

I examined the effect of the controlled food access condition on monkeys’ choice behavior in terms of equivalence relations by analyzing the number of the split choices. If the preferences of food items are close to each other when monkeys are sated (i.e., subjective values of items are close), the number of split choices would increase. Aggregated data across monkeys showed that the number of split choices was almost significantly different between the three food access conditions (sated: 52/120 (43.3%); non-sated: 52/120 (43.3%); hungry: 36/120 (30%), Chi-squared test, P = 0.0502). However, individual-based data analysis showed that among the total 20 choices, the number of the split choices was not significantly different between the three food access conditions (sated: 8.7 ± 1.98 (43.5%); non-sated: 8.7 ± 1.22 (43.5%); hungry: 6.0 ± 1.03 (30%), one-way ANOVA, P = 0.36). Thus, the monkeys’ split choices driven by the individual food preferences might be or might not be affected by hunger states.

## Discussion

In the present study, I examined three issues regarding how hunger states affect economic decision making of marmoset monkeys. First, monkeys’ preferences for food items did not appear to differ according to the distinct hunger states. Second, despite such non-changeable preferences, transitivity violations decreased when monkeys were hungry. Third, when monkeys were hungry, the choice of dominating items sharply increased as the difference of the preference scores increased. These results suggest that hunger decreases the level of stochasticity in the process of decision making and biased animals to maximize their benefits more consistently.

Transitivity is a basic component of rational decision making in economics^34^. It follows from the basic assumption in economics that a chooser assigns values to options according to his internal preference ranking of items (i.e., utility), and uses these to make a choice that maximizes benefits. Transitivity of preferences has been previously measured in humans, honey bees, and birds, using a standard economic procedure, i.e., binary choice testing for item pairs^10, 16–20^ as was employed in this study. In these previous studies, animals are not completely transitive, but are stochastically transitive to some degree. The “stochastic transitivity” in economic theory models the transitivity of preferences in a probabilistic manner. An example of such a condition is as follows: if a chooser is more likely to choose item A than B from {a, b} and more likely to choose B than C from {b, c}, then the chooser will be more likely to choose A than C (i.e., [P(a,b) > 0.5, P(b,c) > 0.5] → [P(a,c) > 0.5]; “P(a,b) > 0.5” means that the probability of choosing item A over item B is more than 50 percent)^34^. Previous study in honey bees has found that majority of the tested bees were stochastically transitive, but some individuals violate stochastic transitivity (i.e., [P(a,b) > 0.5, P(b,c) > 0.5], but [P(a,c) < 0.5])^35^. While these previous studies examined the transitivity violation in great detail, the effect of internal physiological states on transitivity – hunger and thirst – has received little attention.

In the present study, hungry monkeys showed almost no transitivity violations and nearly perfect transitivity: cyclic orders in triples only occurred 5% of the time, equivalent to the level of human choosers in a previous neuroscientific study (4%)^31^. The degree of stochasticity in their preferences increased when they were not hungry (about 15% of the time). This result suggests that the degree of stochasticity changes depending on hunger state.

What process of economic decision making yields this change in stochasticity? The present results suggest that hunger state affects the comparison process of items’ values for choosing items. Monkeys’ choices of dominating items were more sensitive to preference score differences when they were hungry (Fig. 4b). Namely, monkeys choose higher values options more deterministically and seemed to make more efficient maximization when they were hungry. One possible interpretation of this effect is that monkeys explored food items when they were not hungry, but exploited the knowledge of the food values when they were hungry, which is known as the exploration and exploitation dilemma in a dynamic environment^36^. However, since all monkeys were familiar with the food items used in the experimental testing, monkeys may have maximized their income when they were hungry and re-explored the value of food items when they were in a good energetic state.

Another possible explanation is that the values of food items increase (decrease) when animals are hungry (sated), much like a wealth function in economics, i.e., relationships between wealth level and value a subject feels. In my previous study using thirsty macaques, monkeys became more risk averse as they became poorer in terms of water^37, 38^. Moreover, there is some evidence that hungry people make financial decisions differently from sated people^4, 6, 39^, and it is widely acknowledged that less wealthy people make different decisions about whether or not to accept a particular risk compared to more wealthy people^40–42^. Therefore, it seems likely that both valuation and comparison processes for economic choices are influenced by hunger and thirst in the brain. Theoretically, changes in either of these processes should affect the degree of stochasticity of choice behavior, although monkeys’ preferences for food items in the present study, measured as preference scores, did not appear to differ between hunger states (Fig. 2). Note, however, that in the pairwise choice test employed in the current study, measured preferences were only relative to each other within each hunger state, and that pairwise choices cannot be used to evaluate overall changes of items’ values across states. Rating or betting tests would be required to measure these value changes between states, which have been used in human experiments^4, 6, 28^.

How do animals determine the values of consumable items, and are learned item values remembered when animals make economic choices? Both vertebrates and invertebrates show a preference for items experienced when they were in a hungrier state (i.e., state-dependent valuation^43, 44^). For example, grasshoppers have a preference for food items that were consumed when in a low energetic state over those consumed when sated, and this is true regardless of the hunger state at the time of testing^45^. Under this remembered value mechanism, values of each food item are said to be learned from mesolimbic dopamine signals in a state-dependent manner^46^ and those signals might be preserved across different hunger states. This possibility is partly supported by the current observation that preference scores appeared to be stable across distinct hunger states (Fig. 2). However, the value signals represented in the brain likely change across states; the majority of orbitofrontal cortex neurons, a strong candidate for the valuation center in the primate nervous system, decrease their activity when monkeys are sated^47^. Moreover, some studies have reported that neuronal activity in decision-related brain regions change dramatically depending on hunger and thirst^1, 3^. Further study is required to elucidate how the value signals represented in multiple brain regions are preserved or changed across distinct hunger states, and how these lead to consistent preferences for food items as observed in the present study.

Another principle – sensory specific satiety – postulates that values of a particular food item decrease after subjects are satiated for that specific food item^24^. This food-type specific devaluation is in contrast to an overall decrement of all item values when sated, which suggests that two distinct neural mechanisms may control value signals in the brain. It is possible that an “increase” and “decrease” of hunger/thirst may underlie these distinct neuronal processes. For example, activity in the anterior cingulate cortex decreases just after a thirsty human drinks water, even though hydration levels in the cardiovascular system have yet to change^48^. This suggests that some internal devaluation process suppresses consumption behavior before peripheral feedback signals have arrived to the brain^49^. In contrast, the timescale at which animals become hungry or thirsty is much longer compared to such devaluation processes (more than a couple of hours). In the process of increasing values (with increasing thirst and hunger), peripheral physiological feedback, such as blood osmolality level for thirst^38^ and levels of blood glucose and insulin for hunger^1^ may dominate the process of value computation in the brain.

In the present study, the effect of hunger state on the monkeys’ behavior sometimes showed a lack of statistical significance, especially in the individual-based data analyses. For example, an effect of hunger state was observed on the number of the split choices (close to significant, P = 0.0502) and the number of uneaten trials (P < 0.006) in the aggregated data, while those effects were not detected significantly in the individual-based analyses (P = 0.36 and 0.37 for the number of the split choices and uneaten trials, respectively). If I had sampled a greater number of the trials for each food item pair (only two trials were implemented in the present study), the subtle effect of the hunger state on monkeys’ behavior might have been better detected, even on food item preferences whose effects appeared to be stable in this study.

The common marmoset (*Callithrix jacchus*), a New World monkey, is a recently developed primate model for neuroscience, while the macaque monkey, an Old World monkey, is a long-established primate neuroscience model. The recent advances in visual, auditory, and social neurosciences highlighted the importance of marmoset monkeys as an experimental primate model for modern neuroscience research^50^. The present study additionally showed that the marmoset is a suitable model for studies of economic decisions and underlying neural mechanisms. Further study is required to examine whether the preference changes observed extend to humans and other non-human primates.

In summary, the present study showed that captive marmoset monkeys change their transitivity of preferences for food items in a stochastic manner, depending on hunger state. The preferences of food items appeared to remain stable within hunger states, but hungry monkeys were more sensitive to the difference of food preferences, resulting in a decrease of transitivity violations. These results suggest that hunger decreases the level of stochasticity and increase the degree of economic rationality for efficient outcome maximization.

## Methods

### Subjects

Six common marmosets (*Callithrix jacchus*, three males and three females, mean age 3.3 years, range 2–5) were used. Animals were housed in pair or family cages in a colony of the National center of Neurology and Psychiatry, and were housed in individual cages during the experimental period. Animals received chow and fruit, and had *ad libitum* access to water. All experimental procedures were approved by the Primate Research Committee at the National institute of Neuroscience, NCNP, Japan, and were carried out in accordance with the Guide for the Care and Use of Laboratory Animals (National Research Council of National Academies, USA).

### Behavioral apparatus

The experimental chamber contained two rectangular compartments (Fig. 1a). The experimental chamber was attached to the home cage before each experimental session, and detached once the session had ended. A metal wire sliding door was placed on the side closest to the home cage (Fig. 1a, bottom). An acrylic clear panel was placed at the far side of the compartments where food items were placed during testing. The experimenter was thus able to observe whether animals ate a food item or not.

### General experimental procedure

After habituation to the experimental chamber, subjects were familiarized with the pairwise food choice task (about 40 trials). Thereafter, subjects were tested five days a week for 10 minutes in the afternoon, whereby one 10-minute session was composed of two trials. All experiments were performed between 12:00 and 14:00.

### Controlled food access

In the regular food schedule condition (without experimental testing), animals were fed three times per day (Fig. 1c, Regular). Dry chows were provided in the morning, soft chows at noon after two hours without food, and favorite foods such as fruit, bread, and steamed cakes in the evening. These favorite foods were also used for the pairwise food choice test. All leftover food was removed one hour after the delivery of the morning meal, and no food was available until the noon meal. On days on which experimental testing was performed, one of the following three food conditions was applied: sated, non-sated, and hungry conditions (Fig. 1c). The three food access conditions were blocked (10 consecutive days per condition), and the order of blocks was randomized across animals. In the sated condition, all meals were provided at the same times as in the regular food condition, and testing was conducted one hour after the noon meal. In the non-sated condition, all meals were provided at the same times as in the regular food condition, and testing was conducted before the noon meal. In the hungry condition, the morning meal was not provided and, after approximately four hours without food, testing was conducted at noon.

### Pairwise food choice test

All possible pair combinations of the five food items were tested in the pairwise choice test, except for same food pairs (Fig. 1b). Only one food pair was used for the experimental test per day, resulting in two choice trials of one food pair with left-right alternation. At the beginning of the first trial, two food items were placed at the far end of the two compartments of the experimental chamber attached to the home cage. Monkeys saw the food items through the metal wire sliding door. After one minute, the experimenter opened the metal sliding door. If monkeys made a choice and ate one of the two foods, the other food item was removed. The second trial started one minute after monkeys had finished eating the chosen food item in the first trial, and the location of the food items (left/right) were swapped. If the subject did not eat either of the food items, the same food pair was tested at a later date. All 10 food pairs were tested in each of the three food access conditions, resulting in a total of 60 trials (10 pairs × 2 locations × 3 food access conditions) for each monkey. Note that left-right location of food items was counterbalanced, since it has been reported that some marmosets show spatial preferences^51^.

### Data analysis

Data analysis was conducted using the statistical software “R” (http://www.r-project.org/). Behavioral measures of monkeys were analyzed in two ways: i) data was aggregated for all six monkeys (aggregated data analysis), and ii) data was analyzed individually (individual-based analysis).

The number of times that an item was chosen was counted in each of the three food access conditions for each individual monkey. These numbers were defined as preference scores that inferred preferences for each of the food items. Maximum and minimum preference scores were eight and zero, respectively, in each of the three food access conditions. The following four statistical tests were used to examine whether individual preferences for food items were stable or not across the three food access conditions: (i) A Chi-squared test to compare preference scores between the three conditions in each monkey, with significance set at P < 0.05, (ii) An F-test to compare the variance of the preference scores between the three conditions, with significance set at P < 0.05, (iii) Correlation coefficients for the preference score estimated for each pair of the three food access conditions, and iv) Principal component analysis^32, 52^ to evaluate similarity of the preference scores as interindividual and intraindividual Euclidean distances. Although the food item preferences were different between individual monkeys, the analysis evaluated the similarity of the preference scores as a pattern between the six monkeys and between the three food access conditions. The intraindividual distance was defined as the Euclidean distances between the three food access conditions in each monkey. The interindividual distance was defined as the Euclidean distances between the six subjects. The interindividual and intraindividual differences of the Euclidean distances were compared using a two sample t-test, with significance set at P < 0.05.

In each of the three food access conditions, transitivity violation was detected as the number of cyclic orders as in the traditional economic analysis. Perfectly consistent hypothetical choosers would show no cyclic orders, and so if item A was preferred to item B and item B was preferred to item C, item A was also preferred to item C. If preferences are not transitive, cyclic orders would be observed frequently, i.e. inconsistent choosers show cyclic orders: item A was preferred to item B and item B was preferred to item C, but item C was preferred to item A.

During the pairwise choice test in this study, a total number of triples was _5_C_3_ × 2^3^, i.e., all possible combination of three items out of five items (_5_C_3_) and each pairwise comparison of three items were made twice (2^3^):

1 $$(\begin{array}{l}5\\ 3\end{array})\times {2}^{3}=\frac{5!}{3!(5-3)!}\times {2}^{3}=80$$

The total number of all possible combinations equal to or larger than triples was ${}_{5}{\rm{C}}_{3}\times {2}^{3}+{}_{5}{\rm{C}}_{4}\times {2}^{4}+{}_{5}{\rm{C}}_{5}\times {2}^{5}:$

2 $$(\begin{array}{l}5\\ 3\end{array})\times {2}^{3}+(\begin{array}{l}5\\ 4\end{array})\times {2}^{4}+(\begin{array}{l}5\\ 5\end{array})\times {2}^{5}=192$$

The number of transitivity violations was counted as the number of cycles among 20 pairwise choices made in each of the three food access conditions. The number of violations in each of the three food access conditions was compared using a one-way ANOVA, with significance set at P < 0.05.

To examine the relationship between the transitivity violation and the preference score, I calculated the percent choices of the dominating items according to the preference score differences. Dominating items were defined as the items chosen over another twice (i.e., 100% of the time). If the choice of dominating item is frequent, the transitivity violation decreases, as shown in perfectly consistent hypothetical choosers. Since there is no way to predict indifference using only pairwise comparisons, I analyzed the percent choice of dominating food items. The probability *P* of choosing dominating items was analyzed using a generalized linear model with binominal distribution. The relation between *P* and *Z* was given by the following logistic function:

3 $$P=\frac{1}{1+{e}^{-z}}$$

4 $$Z={\alpha }+{\beta }_{1}\times (P{S}_{A}-P{S}_{B})+{\beta }_{2}\times {\rm{FAC}}+{\beta }_{3}\times {\rm{interaction}}$$

where α is the intercept, *PS*_*A*_ and *PS*_*B*_ are the preference scores of item A and B in a food pair, respectively, FAC represents the three food access conditions, and “interaction” is the interaction between the difference of preference scores and the food access conditions. If the *β*_*2*_ was not 0 at P < 0.05, I concluded that the choice of the dominating items depended on the food access conditions. Note that, when the difference score was 0, the percentage of choosing dominant items was defined as the percentage of non-split choices (i.e., dominating choices).

### Data Availability Statement

All relevant data are within the paper.

## References

[1] Simon SA, de Araujo IE, Gutierrez R, Nicolelis MA (2006). The neural mechanisms of gustation: a distributed processing code. Nat Rev Neurosci.

[2] Bourque CW (2008). Central mechanisms of osmosensation and systemic osmoregulation. Nat Rev Neurosci.

[3] de Araujo IE (2006). Neural ensemble coding of satiety states. Neuron.

[4] Symmonds M, Emmanuel JJ, Drew ME, Batterham RL, Dolan RJ (2010). Metabolic state alters economic decision making under risk in humans. PLoS One.

[5] Stephens, D. & Krebs, J. *Foraging Theory* (Princeton Univ. Press, New Jersey, 1986).

[6] Levy DJ, Thavikulwat AC, Glimcher PW (2013). State dependent valuation: the effect of deprivation on risk preferences. PLoS One.

[7] Kacelnik A, El Mouden C (2013). Triumphs and Trials of the Risk paradigm. Animal Behaviour.

[8] Brito e Abreu F, Kacelnik A (1999). Energy budgets and risk-sensitive foraging in starlings. Behavioral Ecology.

[9] Caraco T, Martindale S, Whitham TS (1980). An empirical demonstration of risk-sensitive foraging preferences. Animal Behaviour.

[10] Tversky A (1969). Intransitivity of preferences. Psychological Review.

[11] Von Neumann, J. & Morgenstern, O. *Theory of Games and Economic Behavior*. (Princeton Univ. Press, New Jersey, 1944).

[12] Samuelson PA (1950). The Problem of Integrability in Utility Theory. Economica.

[13] Houthakker HS (1950). Revealed Preference and the Utility Function. Economica.

[14] Savage, L. J. *The Foundations of Statistics* (John Wiley and Sons, New. York, 1954).

[15] Kacelnik, A. Meanings of rationality. *Rational Animals*? (ed. S. Hurley & M. Nudds) (Oxford University Press, Oxford, 2006).

[16] Loomes G, Starmer C, Sugden R (1991). Observing Violations of Transitivity by Experimental Methods. Econometrica.

[17] Shafir S, Waite TA, Smith BH (2002). Context-dependent violations of rational choice in honeybees (Apis mellifera) and gray jays (Perisoreus canadensis). Behavioral Ecology and Sociobiology.

[18] Bateson M, Healy SD, Hurly TA (2002). Irrational choices in hummingbird foraging behaviour. Animal Behaviour.

[19] Latty T, Beekman M (2011). Irrational decision-making in an amoeboid organism: transitivity and context-dependent preferences. Proc Biol Sci.

[20] Grether DM, Plott CR (1979). Economic theory of choice and the preference reversal phenomenon. Am. Econ. Rev..

[21] Loomes G, Starmer C, Sugden R (1991). Observing violations of transitivity by experimental methods. Econometrica.

[22] Rolls BJ, Rolls ET, Rowe EA, Sweeney K (1981). Sensory specific satiety in man. Physiol Behav.

[23] Dickinson A, Balleine B (1994). Motivational control of goal-directed action. Animal Learning and Behavior.

[24] Baxter MG, Murray EA (2002). The amygdala and reward. Nat Rev Neurosci.

[25] Murray EA, Izquierdo A (2007). Orbitofrontal cortex and amygdala contributions to affect and action in primates. Ann N Y Acad Sci.

[26] Rudebeck PH (2008). Frontal cortex subregions play distinct roles in choices between actions and stimuli. J Neurosci.

[27] Rolls ET (2015). Taste, olfactory, and food reward value processing in the brain. Prog Neurobiol.

[28] Hoefling A, Strack F (2010). Hunger induced changes in food choice. When beggars cannot be choosers even if they are allowed to choose. Appetite.

[29] Kral TV (2006). Effects on hunger and satiety, perceived portion size and pleasantness of taste of varying the portion size of foods: a brief review of selected studies. Appetite.

[30] Ueland O, Cardello AV, Merrill EP, Lesher LL (2009). Effect of portion size information on food intake. J Am Diet Assoc.

[31] Levy I, Lazzaro SC, Rutledge RB, Glimcher PW (2011). Choice from non-choice: predicting consumer preferences from blood oxygenation level-dependent signals obtained during passive viewing. J Neurosci.

[32] Bennett JF, Hays WL (1960). Multidimensional Unfolding: Determining the Dimensionality of Ranked Preference Data. Psychometrika.

[33] Wichmann FA, Hill NJ (2001). The psychometric function: I. Fitting, sampling, and goodness of fit. Percept Psychophys.

[34] Fishburn PC (1973). Binary choice probabilities: on the varieties of stochastic transitivity. Journal of Mathematical Psychology.

[35] Shafir S (1994). Intransitivity of preferences in honey bees: support for ‘comparative’ evaluation of foraging options. Animal Behaviour.

[36] Daw ND, O’Doherty JP, Dayan P, Seymour B, Dolan RJ (2006). Cortical substrates for exploratory decisions in humans. Nature.

[37] Yamada H, Tymula A, Louie K, Glimcher PW (2013). Thirst-dependent risk preferences in monkeys identify a primitive form of wealth. Proc Natl Acad Sci USA.

[38] Yamada H, Louie K, Glimcher PW (2010). Controlled water intake: a method for objectively evaluating thirst and hydration state in monkeys by the measurement of blood osmolality. J Neurosci Methods.

[39] Emmanuel, J. *et al*. Gamble on a Full Stomach: Monetary Risk-Taking Is Altered by Metabolic State in Normal-Weight Human Male Subjects. In *ENDOCRINE REVIEWS* (San Diego, CA, 2010).

[40] Tanaka T, Camerer CF, Nguyen Q (2010). Poverty, Politics, and Preferences: Field Experiments and Survey Data from Vietnam. American Economic Review.

[41] Yesuf M, Bluffstone RA (2009). Poverty, Risk Aversion, and Path Dependence in Low-Income Countries: Experimental Evidence from Ethiopia. American Journal of Agricultural Economics.

[42] von Gaudecker H-M, van Soest A, Wengström E (2011). Heterogeneity in Risky Choice Behaviour in a Broad Population. American Economic Review.

[43] Revusky SH (1967). Hunger level during food consumption: Effects on subsequent preference. Psychonomic Science.

[44] Capaldi ED, Myers DE (1982). Taste preferences as a function of food deprivation during original taste exposure. Animal Learning and Behaviour.

[45] Pompilio L, Kacelnik A, Behmer ST (2006). State-dependent learned valuation drives choice in an invertebrate. Science.

[46] Papageorgiou GK, Baudonnat M, Cucca F, Walton ME (2016). Mesolimbic Dopamine Encodes Prediction Errors in a State-Dependent Manner. Cell Rep.

[47] Critchley HD, Rolls ET (1996). Hunger and satiety modify the responses of olfactory and visual neurons in the primate orbitofrontal cortex. J Neurophysiol.

[48] Egan G (2003). Neural correlates of the emergence of consciousness of thirst. Proc Natl Acad Sci USA.

[49] Houpt TR, Yang-Preyer H, Geyer J, Norris ML (1999). A rapid feedback signal is not always necessary for termination of a drinking bout. Am J Physiol.

[50] Tokuno H, Watson C, Roberts A, Sasaki E, Okano H (2015). Marmoset neuroscience. Neurosci Res.

[51] Adriani W, Romani C, Manciocco A, Vitale A, Laviola G (2013). Individual differences in choice (in)flexibility but not impulsivity in the common marmoset: an automated, operant-behavior choice task. Behav Brain Res.

[52] Sinn DL, Moltschaniwskyj NA (2005). Personality traits in dumpling squid (Euprymna tasmanica): context-specific traits and their correlation with biological characteristics. J Comp Psychol.

